# Spatial Structure Explains Morphological Variation Better Than Climatic Gradients in the South American Rattlesnake (*Crotalus durissus*)

**DOI:** 10.1002/ece3.73298

**Published:** 2026-03-15

**Authors:** Mileny Otani, Henrique Caldeira Costa, Cláudio Henrique Zawadzki, Diego J. Santana

**Affiliations:** ^1^ Museu de Zoologia João Moojen, Departamento de Biologia Animal Universidade Federal de Viçosa Viçosa Minas Gerais Brazil; ^2^ Programa de Pós‐Graduação em Biodiversidade e Conservação da Natureza Universidade Federal de Juiz de Fora Juiz de Fora Minas Gerais Brazil; ^3^ Núcleo de Pesquisas em Limnologia, Ictiologia e Aquicultura (Nupélia) Universidade Estadual de Maringá Maringá Paraná Brazil; ^4^ Negaunee Integrative Research Center and Keller Science Action Center The Field Museum of Natural History Chicago Illinois USA

**Keywords:** latitudinal gradient, macroecological patterns, sexual dimorphism, venomous snakes, Viperidae

## Abstract

Morphological variation in vertebrates is often shaped by geographic and climatic factors, yet the applicability of broad‐scale ecogeographical rules remains debated, particularly in ectotherms. We investigated how climatic gradients and spatial structure influence body size in the South American rattlesnake ( *Crotalus durissus* ), a widely distributed species across diverse ecoregions. We analyzed morphometric data from 132 adult individuals, integrating 19 bioclimatic variables from WorldClim and geographic coordinates for each collection site. Body size variation was summarized using principal component analysis, and spatial autocorrelation was explicitly incorporated into the analytical framework. After accounting for spatial structure, climatic variables were not significant predictors of body size in either sex. Instead, males exhibited a latitudinal spatial trend, whereas females showed a structured southwestward decrease in size. These results indicate that spatial constraints and sex‐specific ecological pressures, rather than contemporary climatic gradients, shape morphological variation in *C. durissus* . Our findings highlight the importance of incorporating spatially explicit models when evaluating climate–trait relationships and contribute to a more nuanced understanding of morphological evolution in widespread ectothermic species.

## Graphical abstract

We evaluated whether climatic gradients or spatial structure better explain body size variation in the South American rattlesnake ( *Crotalus durissus* ) across its wide geographic range. After explicitly accounting for spatial autocorrelation, climatic variables were not significant predictors of body size in either sex, whereas sex‐specific spatial patterns were detected. These findings indicate that spatial structure, rather than contemporary climatic gradients, plays a predominant role in shaping morphological variation in this widespread ectothermic species.

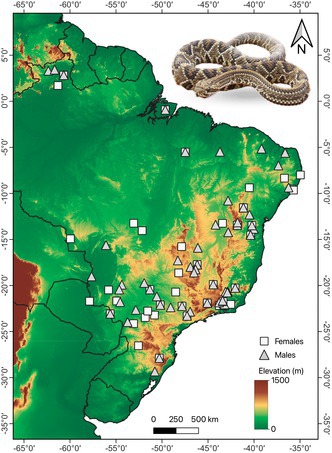

## 1 Introduction

What drives morphological variation in animals across space? This question is central to understanding how organisms respond to environmental pressures and geographic constraints. Body size, in particular, is a key functional trait linked to physiology, behavior, and fitness, and has long been a focus of biogeographical research (Romano and Ficetola 2010; Schiaffini 2016; Lopez et al. 2016; Ahti et al. 2020; Clifton et al. 2020; Roitberg et al. 2020; Romano et al. 2020; Weber et al. 2021; Sebastianelli et al. 2022). Classical ecological rules, such as Bergmann's, Rapoport's, and the theory of island biogeography, have been proposed to explain patterns of body size and distribution, yet their generality across taxa, especially in ectothermic vertebrates, remains contentious (Smith and Brown 2002; Cruz et al. 2005; Boaratti and Silva 2015; Benítez‐López et al. 2021; Caten et al. 2022). At broad geographic scales, climatic gradients are inherently correlated with geographic space, making it difficult to disentangle environmental effects from spatial structure (Borcard et al. 1992; Legendre et al. 2005). Understanding whether climatic gradients or spatial processes are the main forces shaping intraspecific variation is essential for predicting how species will respond to environmental change.

Bergmann's rule predicts that animals are larger in colder climates, usually at higher latitudes (Bergmann 1847; Servino et al. 2024), a pattern widely supported in many endotherm species, such as birds and mammals (Ashton et al. 2000; Ashton 2002; He et al. 2023). However, for ectotherms, these patterns are often inconsistent across different taxonomic groups (Ashton and Feldman 2003; Alcantara et al. 2024). Because they rely on microhabitat temperatures for thermoregulation (Chan et al. 2024; Gardner et al. 2024), some ectotherms, from insects to vertebrates, may not conform to these classical rules (Belk and Houston 2002; Ashton and Feldman 2003; Adams and Church 2008; Alcantara et al. 2024). Furthermore, even in communities where Bergmann's rule has historically been observed, anthropogenic pressures can rapidly erode these latitudinal gradients, leading to functional homogenization of body sizes (Fisher et al. 2010). In fact, body size in ectotherms is often more directly tied to local environmental conditions such as solar radiation, temperature, and moisture availability, which also influence growth rate, mobility, and dispersal (Stevenson 1985; Paaijmans et al. 2013; Burraco et al. 2020). Importantly, testing ecogeographical rules using latitude alone may confound climatic effects with underlying spatial structure, potentially inflating or obscuring true environmental associations (Dormann et al. 2007).

In some cases, morphological divergence among populations follows a clinal pattern, in which greater geographic distances correspond to increased morphological differentiation (Turan 2000; Passos et al. 2005; Allsteadt et al. 2006; Cardini et al. 2007; Costa et al. 2013; Klepsatel et al. 2014; Abreu et al. 2018). Such variation may reflect an isolation‐by‐distance process, especially in species with limited dispersal ability or philopatric behavior (Wright 1943; Avise 2000). In snakes, clinal trends in traits such as pholidosis, body proportions, and coloration have been reported along latitudinal and longitudinal gradients (e.g., Allsteadt et al. 2006; Passos and Fernandes 2008; Mebert 2011; Costa et al. 2013; Watson et al. 2019; Fritz and Ihlow 2022). These patterns highlight how spatial structure can shape phenotypic diversity, even in taxa with broad distributions. However, without explicitly accounting for spatial autocorrelation, it remains challenging to determine whether observed clines reflect environmental adaptation or spatially structured processes independent of environmental gradients.

Snakes, as highly diverse ectothermic vertebrates (Grundler and Rabosky 2021), offer a compelling system to explore these issues. Their elongated bodies, broad ecological diversity, and wide geographic ranges allow for meaningful tests of how morphology varies across space (Caldwell et al. 2015; Martill et al. 2015; Watson et al. 2019). Such variation often appears through phenotypic divergence in response to local environmental conditions (Manier 2004), manifesting as shifts in complex structures like skull shape (Watson et al. 2019) or in aggregate traits such as body size, which may or may not follow classic ecogeographical patterns like Bergmann's rule (Servino et al. 2024). Despite being physiologically constrained by temperature, they can inhabit diverse environments, from arid grasslands and savannas to humid forests and montane habitats (Campbell and Lamar 2004), where morphological characters can serve as vital indicators of evolutionary history and ecological adaptation (Roth‐Monzón et al. 2021).

Some snake species, such as the South American rattlesnake *Crotalus durissus* Linnaeus 1758, occupy a broad range of ecoregions and latitudes. *Crotalus durissus* is widely distributed across South America (Wuster et al. 2005; Nogueira et al. 2019; Arias‐Sosa et al. 2025). It inhabits a variety of environments, including open, human‐altered areas, and even slightly forested and flooded habitats. (Klauber 1984). Its presence across such heterogeneous environmental and spatial gradients makes it an excellent model for testing whether morphological variation is best explained by climatic gradients, as predicted by ecogeographic rules, or by spatial structure independent of climate.

In this study, we investigate how climatic and geographic factors influence morphological variation in *C. durissus* by analyzing morphometric data from individuals across multiple locations within its distribution and integrating these data with climatic and spatial variables. Specifically, we test whether body size variation is more strongly associated with climatic gradients (e.g., temperature and precipitation) in accordance with ecogeographical rules, or with explicit spatial structure, while formally accounting for spatial autocorrelation.

## 2 Materials and Methods

### 2.1 Data Collection

**FIGURE 1:**
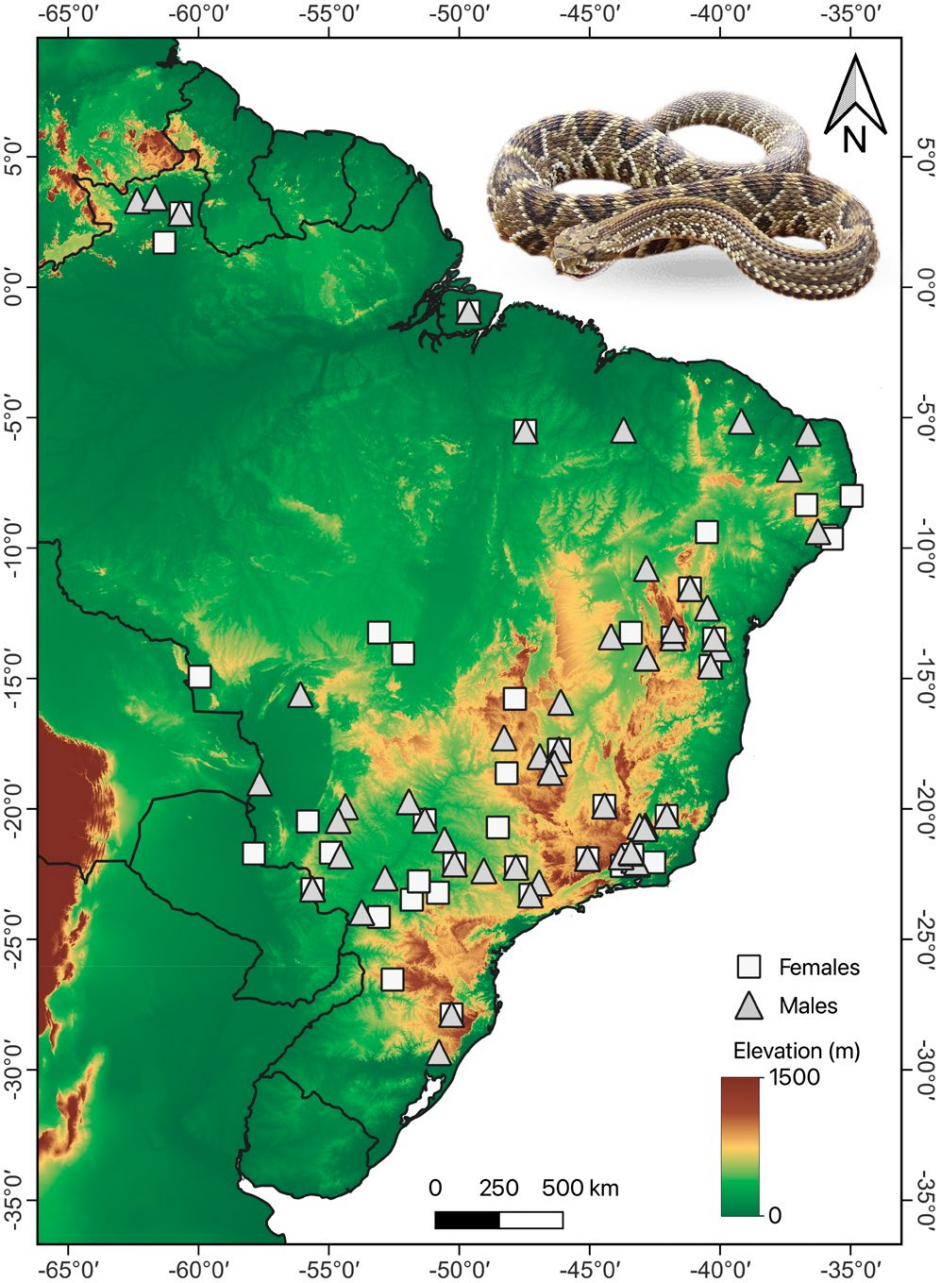
Geographical distribution of the *Crotalus durissus* specimens analyzed in this study. Detailed locality information is shown in Table S1. Highlighted in the map is an individual from the municipality of Alcinópolis, MS, Brazil.

We analyzed 132 adult specimens (80 males and 52 females; Figure 1; Table S1) from six Brazilian zoological collections: Instituto Butantan (IBSP), Museu de Zoologia da Universidade Estadual de Santa Cruz (MZUESC), Museu de Zoologia João Moojen (MZUFV), Museu Nacional do Rio de Janeiro (MNRJ), Museu de Zoologia da Universidade de São Paulo (MZUSP), and the Coleção Zoológica da Universidade Federal de Mato Grosso do Sul (ZUFMS‐REP). The collection dates span from 1916 to 2023 (Table S1), with 77% (101 specimens) of the sampled snakes collected between 1970 and 2015. We selected specimens to maximize geographic coverage across the species' known distribution range, based on their availability in those collections. Our sampling strategy focused on representing diverse regions, including specimens from the Atlantic Forest, Amazonia, Caatinga, and Cerrado biomes (sensu IBGE—Instituto Brasileiro de Geografia e Estatística 2025), as well as ecological transition zones. Geographic coordinates were obtained from collection records; when unavailable, we assigned coordinates using the centroid of the reported municipality/locality in Google Earth Pro software (Google Earth 2025). Maps were prepared in QGIS (version 3.34.1) (QGIS 2024). Sampling records were georeferenced by the authors, and the cartographic base included federal and state limits provided by IBGE (2025). Environmental layers for elevation, mean annual temperature, and mean annual precipitation were obtained from Guimarães (2025). All spatial data were projected using the SIRGAS 2000 geographic coordinate system (EPSG:4674).

We took linear morphometric measurements using a digital caliper (0.01 mm precision) and measuring tape (1 mm precision). Body size at sexual maturity varies substantially among *C. durissus* populations (Shine 1994; Barros et al. 2012; Hoyos et al. 2025). Reported minimum sizes range from approximately 560 to 754 mm in males and from 627 to 760 mm in females, with occasional records of smaller gravid females (Almeida‐Santos 2005; Pucca et al. 2021). Considering this variation, we calculated the mean of the minimum reported maturity sizes and established conservative cut‐offs of 696 mm SVL for males and 764 mm for females to exclude potential juveniles from the analysis. We determined sex based on morphological differences in the caudal region according to the Viperidae‐specific criteria (Bernarde 2012), which compares tail length to width posterior to the cloaca. When there was uncertainty, a longitudinal incision was made at the base of the tail to confirm the presence or absence of hemipenes.

### 2.2 Data Analysis

We first tested for sexual dimorphism using a multivariate analysis of variance (MANOVA), including snout–vent length (SVL), tail length (TL), head length (HL), and head width (HW) as response variables and sex as the predictor. Because significant multivariate differences were detected, all subsequent spatial and climatic models were conducted separately for males and females using *lme4* (Bates et al. 2015).

To explore overall body size variation, we conducted a principal component analysis (PCA) on the four standardized morphological variables (SVL, TL, HL, and HW), pooling individuals from all localities to define a common morphospace. The first principal component (PC1) accounted for 65.9% of the total morphological variation and was strongly positively correlated with all traits, representing a composite body size axis (Figure S1; Table S2). PC1 scores were subsequently used as the response variable in spatial and climatic models.

Climatic data were extracted from the 19 bioclimatic variables (Table S4) available in the WorldClim database version 2.1 (Fick and Hijmans 2017) at a spatial resolution of 30 arc‐seconds (~1 km^2^). These variables represent long‐term climatic normals for the period 1970–2000. Climatic values were extracted at each specimen locality after converting coordinates to spatial simple features (WGS84; EPSG:4326). To reduce dimensionality, we performed a second PCA on the bioclimatic variables. The first component (Dim.1) explained 48.1% of the total climatic variation (Figure S2; Table S3) and was used as a synthetic environmental predictor. The strongest contributors to Dim.1 (bio11, bio09, and bio06) primarily reflected a thermal gradient (Figure S3).

To evaluate geographic structure, we implemented an explicit trend‐surface approach by including latitude and longitude, along with their quadratic terms (Lat^2^ and Long^2^) and interaction term (Lat × Long), as spatial predictors in linear models. For each sex, linear models were fitted with morphological PC1 as the response variable and Dim.1 plus spatial polynomial terms as predictors. Model assumptions were evaluated using diagnostic plots to assess residual normality, homoscedasticity, and linearity.

To quantify spatial autocorrelation, we calculated Moran's *I* using a *k*‐nearest neighbors (*k* = 6) weighting scheme. Moran's *I* was computed for morphological PC1 (overall and separately by sex) and subsequently applied to model residuals to determine whether spatial dependence remained after accounting for climatic and spatial predictors.

All statistical analyses and spatial procedures were conducted in R version 4.5.0 (R Core Team 2024). Principal component analyses were performed using the FactoMineR package (Lê et al. 2008). Climatic raster data were processed and extracted using the raster package (Hijmans 2025), and spatial objects were handled using the sf package (Pebesma 2018). Spatial autocorrelation analyses (Moran's *I*) were implemented using the spdep package (Bivand et al. 2005). Model diagnostics were evaluated using the performance package (Lüdecke et al. 2021). Data manipulation and visualization were performed using tidyverse (Wickham et al. 2019), including ggplot2, and PCA visualizations were generated with factoextra (Kassambara and Mundt 2020).

## 3 Results

The morphological variation is summarized in Table 1. For the morphological traits, PC1 explains 65.9% of the total variation, and PC2 explains 18.8%, together accounting for 84.8% of the morphological variance. All four variables (SVL, TL, HL, and HW) exhibited strong positive loadings on PC1, indicating that PC1 is a robust proxy for overall body size.

**TABLE 1:** Morphometric measurements (in mm) of *Crotalus durissus* specimens analyzed. Values are presented as ranges and means ± standard deviations (SD) for all specimens and separately for females and males.

	All		Females		Males	
	Range	Mean ± SD	Range	Mean ± SD	Range	Mean ± SD
Snout‐vent length	710–1375	915 ± 134	800–1375	919.5 ± 127.3	710–1305	879.5 ± 136.7
Tail length	46–182	86 ± 24	50–129	69 ± 15.6	46–182	97.5 ± 21.4
Head length	36–87	50 ± 8.5	36–87	50 ± 8.8	39–82	50 ± 8.3
Head width	21–69	39 ± 8.8	22–69	40 ± 9.3	21–64	38 ± 8.5

**FIGURE 2:**
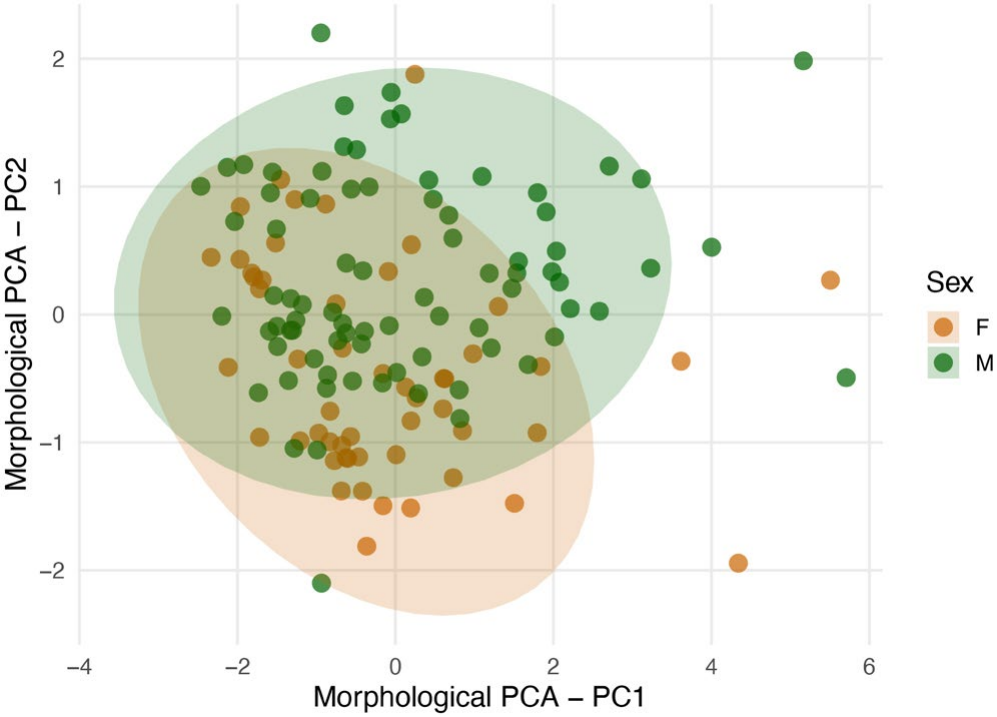
Sexual differences between males (green) and females (orange) of *Crotalus durissus* , based on a principal component analysis (PCA) of four morphometric traits. PC1 explains 65.9% of the total variation and PC2 explains 18.8%, together accounting for 84.8% of the morphological variance. Shaded ellipses represent 95% confidence intervals. Although male and female morphospaces partially overlap, the difference is statistically significant (MANOVA, *p* < 0.0001), supporting the presence of sexual dimorphism.

Regarding sexual dimorphism, male and female morphospaces partially overlap, but the difference between sexes is statistically significant (MANOVA: Wilks' *λ* = 0.3186, *F*_4_,_127_ = 67.91, *p* < 2.2 × 10^−16^; Figure 2), with females bigger than males (Table 1). Univariate tests revealed that males possessed significantly longer tails than females (TL: *F*_1_,_130_ = 68.31, *p* = 1.41 × 10^−13^), whereas females tended to reach larger snout–vent lengths, although this difference was marginally non‐significant (SVL: *F*_1_,_130_ = 3.15, *p* = 0.078). No significant differences were detected in head length (HL: *F*_1_,_130_ = 0.11, *p* = 0.74) or head width (HW: *F*_1_,_130_ = 0.57, *p* = 0.45). Detailed comparisons are provided in Table S5.

**FIGURE 3:**
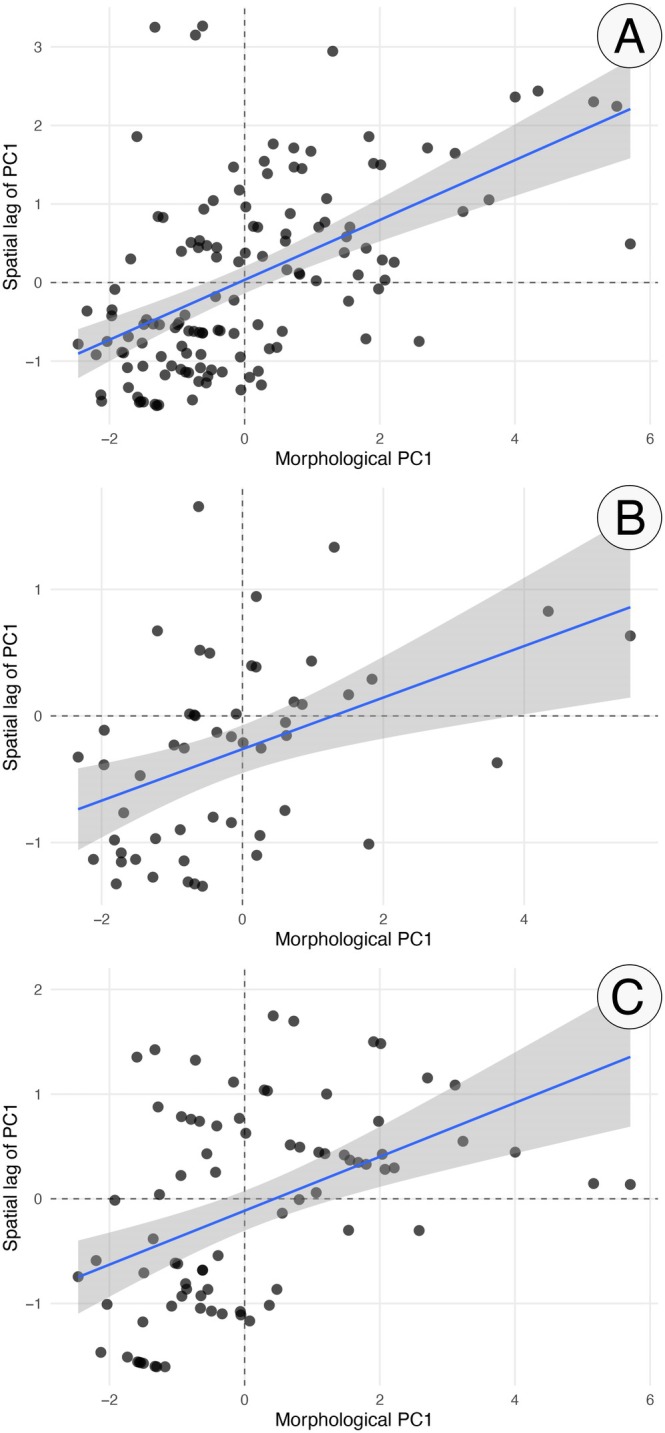
Moran scatterplots illustrating spatial autocorrelation in morphological body size (PC1) of *Crotalus durissus* . (A) All individuals combined; (B) females; (C) males. Each point represents a specimen, with the *x*‐axis showing individual PC1 scores and the *y*‐axis representing the spatial lag of PC1 based on a *k*‐nearest neighbors weighting scheme (*k* = 6). The solid line indicates the fitted linear relationship, and the shaded area represents the 95% confidence interval. Dashed lines denote zero values on both axes. Positive slopes indicate significant positive spatial autocorrelation, reflecting spatial structuring of body size across the species' distribution.

Spatial autocorrelation analyses revealed significant positive Moran's *I* values for morphological PC1 overall (*I* = 0.381, *p* < 0.001), as well as when males (*I* = 0.258, *p* < 0.001) and females (*I* = 0.204, *p* = 0.003) were analyzed separately, indicating spatial structure in body size across the species' distribution (Figure 3).

Spatially controlled linear models revealed no significant association between body size (PC1) and climatic variation (Dim.1) in either sex. In males, Dim.1 showed no significant effect (*t* = −0.902, *p* = 0.370). None of the spatial polynomial terms were individually significant predictors (all *p* > 0.24), although the overall model was statistically significant (*F*_6_,_73_ = 2.61, *p* = 0.024; adjusted *R* ^2^ = 0.109; see Figure S4).

In females, Dim.1 also showed no significant association with body size (*t* = −0.587, *p* = 0.560). However, significant spatial structure was detected through geographic polynomial terms, with longitude (*t* = 2.36, *p* = 0.023) and longitude^2^ (*t* = 2.06, *p* = 0.046) contributing significantly to the model (*F*_6_,_45_ = 5.31, *p* < 0.001; adjusted *R* ^2^ = 0.337; see Figure S5). Model diagnostics indicated adequate fit and no violations of normality or homoscedasticity assumptions.

Residual spatial autocorrelation analyses revealed contrasting patterns between sexes. In females, Moran's *I* calculated on model residuals was not significant (*I* = −0.007, *p* = 0.38), indicating that spatial structure was adequately accounted for. In males, however, residuals still exhibited weak but significant spatial autocorrelation (*I* = 0.105, *p* = 0.029), suggesting that some spatial dependence remains unexplained.

## 4 Discussion

Spatial constraints and sex‐specific selective pressures, rather than climatic gradients, are the primary drivers of body size variation in *C. durissus* . Climatic variables had no significant effect on morphology in either sex. After explicitly accounting for spatial structure in our models, climatic gradients remained non‐significant, reinforcing that large‐scale morphology is not directly associated with contemporary climatic variation. Instead, we identified distinct sex‐specific spatial patterns: male body size showed a spatial association structured along a latitudinal component of the spatial trend surface, whereas female body size exhibited a southwestward decrease. Importantly, these associations were detected after controlling for spatial autocorrelation, indicating that they do not simply reflect unmodeled spatial structure. These results suggest that spatial structuring and distinct ecological roles influence size more than direct environmental temperature. The observed sexual dimorphism, with females larger and males with longer tails, is consistent with previous studies (e.g., Barros et al. 2012; Hoyos et al. 2025) and underscores the need for sex‐separated analyses in wide‐ranging snakes.

Because our sample includes individuals from across most of the geographic range of *C. durissus* , our results might reflect a broader pattern of sexual dimorphism within the species. This could obscure local sexual differences. For instance, our results differ from those reported for *C. d. terrificus*, where males are generally larger than females (Almeida‐Santos et al. 1999). Conversely, no significant sexual size dimorphism has been observed in *C. d. cascavella* (Barros et al. 2012) or *C. d. ruruima* (Pucca et al. 2021). Importantly, we found that males have longer tails, a common sexually dimorphic feature in snakes linked to reproductive anatomy (Shine et al. 1999).

The clinal pattern in female body size, distinct from the spatial trend observed in males, raises questions about the underlying sex‐specific ecological pressures. Thermoregulatory needs during gestation could influence female dispersal and spatial distribution in *C. vegrandis* (Moniz et al. 2024). If similar behavioral thermoregulation constraints exist in pregnant *C. durissus* females, they could limit dispersal or favor occupancy of specific thermal niches, potentially shaping the spatial structuring of morphology observed in this sex. This hypothesis aligns with the idea that female ecology in viviparous snakes may be strongly governed by reproductive constraints (Lourdais et al. 2004). While our study did not test dispersal or gestational behavior, future research integrating spatial ecology, thermoregulatory behavior, and reproductive status across the species' range could elucidate whether such mechanisms contribute to the sex‐specific spatial patterns in morphology reported here.

*Crotalus durissus* shows a preference for microhabitats under shrubs, which offer shade and thermal refuge (Tozetti and Martins 2008), and its range expansion into altered areas is associated with the availability of pastures and vegetation cover that mimic Cerrado conditions (Guerra et al. 2023). Furthermore, the seasonal reproductive cycle and viviparity in *C. durissus* , with a clear biennial cycle in females, may underscore a dependence on stable environmental conditions for sperm storage and embryogenesis (Almeida‐Santos and Orsi 2002; Matayoshi et al. 2018). Collectively, this evidence suggests that gravid females prioritize thermoregulation over dispersal, selecting habitats that enhance survival and reproductive success. This can lead to spatial and behavioral differences compared to males and nonreproductive females. Such spatially constrained reproductive ecology may help explain why female morphology exhibited structured spatial variation independent of climatic predictors.

Our results reinforce previous evidence that classical ecogeographical rules, such as Bergmann's, may have limited applicability to ectotherms (Belk and Houston 2002; Ashton and Feldman 2003; Adams and Church 2008; Alcantara et al. 2024). While Bergmann's rule predicts larger body sizes in colder environments, we found no significant relationship between climatic gradients and body size in either sex. Moreover, because our analyses explicitly accounted for spatial autocorrelation, the absence of climatic effects cannot be attributed to spatial confounding. This is consistent with studies across squamates that often detect no pattern (or even inverse trends) with latitude (e.g., Ashton 2002; Reed 2003; Feldman and Meiri 2014). *Crotalus durissus* , with its broad ecological range and thermoregulatory behavior, may deviate from these rules due to high environmental tolerance and plasticity. Indeed, recent phylogeographic data underscore this adaptability, showing that *C. durissus* rapidly colonized diverse South American ecoregions, ranging from arid trans‐Andean shrublands to the Amazonian basin (Arias‐Sosa et al. 2025).

The lack of a clear climatic signal suggests that phenotypic plasticity may be more influential genetic adaptation across climatic gradients in this species. In reptiles, including squamates, traits like body size can be highly plastic in response to developmental temperature and resource availability (Noble et al. 2018; While et al. 2018). For a wide‐ranging and generalist snake like *C. durissus* , such plasticity could buffer populations against local selection pressures, allowing viable body sizes across diverse habitats without strong climatic clines. Under this scenario, spatial structuring may reflect demographic history, dispersal dynamics, or localized ecological pressures rather than direct climatic selection.

Nevertheless, the interpretation of these eco‐morphological patterns must account for the broad temporal scale of our dataset. Our study is based on museum specimens collected over a 107‐year period (1916–2023), whereas the environmental layers used represent long‐term climatic averages (1970–2000). Environmental layers were not aligned with the exact year of specimen collection, since 30‐year climatic normals better represent long‐term selective regimes than yearly climatic fluctuations. However, recent evidence indicates that snake body size and growth rates can shift substantially over ecological timescales in response to climate change (Elmberg et al. 2024). Such temporal dynamics may introduce noise when long‐term morphological datasets are analyzed against static climatic variables. Additionally, *C. durissus* has experienced a rapid range expansion in South America, largely driven by land‐use change and deforestation (Guerra et al. 2023). Although we found no support for climate‐driven size variation under current conditions, future temporal analyses may reveal dynamic responses to ongoing anthropogenic climate warming.

Although the spatial association observed in males followed a latitudinal axis, climatic predictors were not significant, indicating that this pattern does not represent a classical Bergmann response driven by temperature. Instead, the latitudinal trend likely reflects spatially structured ecological pressures unrelated to contemporary climatic gradients. A more plausible explanation for the patterns observed in males is the starvation resistance hypothesis (Calder 1984), which posits that a larger body confers greater resistance to food scarcity, potentially providing adaptive advantages in highly seasonal environments. This may be particularly relevant for males due to the high energetic costs of active mate‐searching behaviors. Evidence from other viperids suggests that male body size may be more tightly coupled with survival optima dictated by local prey characteristics, whereas female size may be shaped more strongly by fecundity selection (Forsman 1991).

Furthermore, habitat structure and prey availability vary significantly across the range of *C. durissus* , especially considering its recent expansion from the “dry diagonal” into forested biomes. Such anthropized environments often provide abundant rodent prey (Tozetti and Martins 2008). Spatial heterogeneity in resource availability and landscape structure may therefore generate the observed morphological gradients without invoking direct climatic causation. In addition, the wide ecological range of *C. durissus* and its potential for local adaptation make this species a valuable model for studying morphological evolution. Its occurrence across distinct habitats, from arid savannas to humid forests and high‐altitude rocky fields, reflects considerable ecological plasticity (Klauber 1984; Tozetti and Martins 2008). However, growing evidence suggests that *C. durissus* may represent a species complex (Vanzolini and Calleffo 2002; Wuster et al. 2005; Carbajal‐Márquez et al. 2020; Arias‐Sosa et al. 2025). In this context, morphological variation may reflect not only ecological gradients but also underlying genetic structure. For example, phylogenetic relatedness has been shown to explain a significant portion of body size variation in viperids (Terribile et al. 2009), and high levels of gene flow and genetic diversity have been detected in some wide‐ranging snake taxa (Clark et al. 2008; Ursenbacher et al. 2008). Future integrative studies combining morphometric, ecological, and genomic data could clarify whether the patterns observed here reflect lineage divergence, local adaptation, or phenotypic plasticity.

Overall, our study demonstrates that spatial structure, rather than climatic gradients, explains broad‐scale morphological variation in *C. durissus* , and that sex‐specific patterns may reflect differing ecological and reproductive pressures. By explicitly incorporating spatial autocorrelation into our analytical framework, we provide a more robust evaluation of climate–morphology relationships than approaches relying solely on latitudinal proxies. These findings underscore the importance of integrating spatial statistics, ecological context, and evolutionary perspectives to better understand trait variation in widespread ectothermic species.

## Author Contributions

**Mileny Otani:** conceptualization (equal), data curation (equal), formal analysis (equal), investigation (equal), methodology (equal), visualization (equal), writing – original draft (equal), writing – review and editing (equal). **Henrique Caldeira Costa:** conceptualization (equal), validation (equal), writing – original draft (equal), writing – review and editing (equal). **Cláudio Henrique Zawadzki:** supervision (equal), writing – review and editing (equal). **Diego J. Santana:** conceptualization (lead), formal analysis (lead), investigation (equal), methodology (equal), writing – review and editing (equal).

## Funding

This study was supported by CAPES (Coordenação de Aperfeiçoamento de Pessoal de Nível Superior, Brazil) providing a scholarship to M.O. (Finance Code 001). CNPq (Conselho Nacional de Desenvolvimento Científico e Tecnológico, Brazil) provided a research fellowship to D.J.S. (CNPq 311284/2023‐0).

## Conflicts of Interest

The authors declare no conflicts of interest.

## Supporting information


**Data S1:** ece373298‐sup‐0001‐Supinfo.docx.

## Data Availability

The data and R scripts supporting the results of this study are available in the Figshare repository at https://doi.org/10.6084/m9.figshare.31415534.
