# Supplementary material for: Spatial Structure Explains Morphological Variation Better Than Climatic Gradients in the South American Rattlesnake (Crotalus durissus)
Source: Ecol Evol. 2026 Mar 15;16(3):e73298. doi: 10.1002/ece3.73298 (PMC13093689; doi:10.1002/ece3.73298)
Supplement: Supplementary file 1 — Data S1: ece373298‐sup‐0001‐Supinfo.docx. [file ECE3-16-e73298-s001.docx]

**SUPPLEMENTAR MATERIAL**

**Spatial structure explains morphological variation better than climatic gradients in the South American Rattlesnake (*Crotalus durissus*)**

Mileny Otani^1^ | Henrique C. Costa^2^ | Cláudio H. Zawadzki^3^ | Diego J. Santana^4^

^1^Museu de Zoologia João Moojen, Departamento de Biologia Animal, Universidade Federal de Viçosa, Viçosa, Minas Gerais, Brasil | ^2^Programa de Pós-Graduação em Biodiversidade e Conservação da Natureza, Universidade Federal de Juiz de Fora, Juiz de Fora, Minas Gerais, Brasil | ^3^Núcleo de Pesquisas em Limnologia, Ictiologia e Aquicultura (Nupélia), Universidade Estadual de Maringá, Maringá, Paraná, Brasil | ^4^Negaunee Integrative Research Center and Keller Science Action Center, The Field Museum of Natural History, Chicago, Illinois, USA

**FIGURE SM1 |** Eigenvalues and percentage of variance explained by each principal component of the morphological PCA (SVL, TL, HL, HW). PC1 explains 65.9% of the total variation and PC2 explains 18.8%. Blue bars indicate the percentage of variance explained by each principal component, and the black line represents the corresponding eigenvalues.

**FIGURE SM2 |** Eigenvalues and percentage of variance explained by each principal component of the climatic PCA based on 19 WorldClim bioclimatic variables. Dim.1 explains 48.1% of the total climatic variation. Blue bars indicate the percentage of variance explained by each principal component, and the black line represents the corresponding eigenvalues.

**
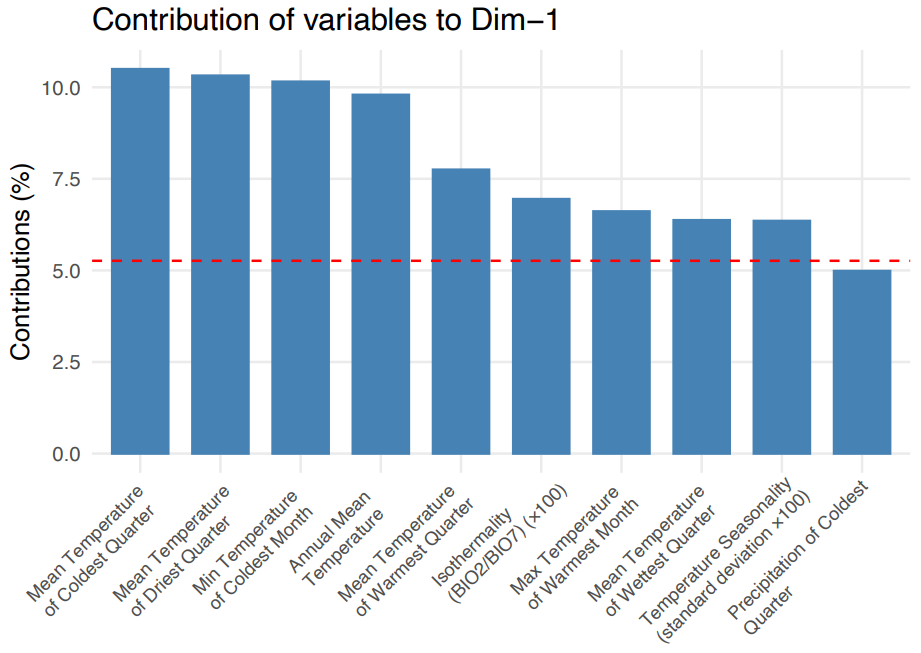
FIGURE SM3 |** Contribution of the ten most influential bioclimatic variables (WorldClim v2.1) to the first component (Dim.1) in the climatic PCA. Variables bio11 (mean temperature of coldest quarter), bio09 (mean temperature of driest quarter), and bio06 (minimum temperature of coldest month) show the strongest contributions, indicating that Dim.1 primarily reflects a thermal gradient. The red dashed line indicates the expected average contribution threshold; bars above it identify variables with a significant influence on the component's structure.

**FIGURE SM4 |** Moran scatterplot of residuals from the spatially controlled linear model for *Crotalus durissus* males. A weak but significant positive spatial autocorrelation remains (*I* = 0.105, *p* = 0.029), indicating residual spatial structure not fully captured by the polynomial trend surface. Each point represents a specimen, with the x-axis showing individual PC1 scores and the y-axis representing the spatial lag of PC1. The solid line indicates the fitted linear relationship, and the shaded area represents the 95% confidence interval. Dashed lines denote zero values on both axes.

**FIGURE SM5 |** Moran scatterplot of residuals from the spatially controlled linear model for *Crotalus durissus* females. Spatial autocorrelation is no longer significant (*I* = –0.007, *p* = 0.38), indicating that spatial structure was adequately accounted for in the model. Each point represents a specimen, with the x-axis showing individual PC1 scores and the y-axis representing the spatial lag of PC1. The solid line indicates the fitted linear relationship, and the shaded area represents the 95% confidence interval. Dashed lines denote zero values on both axes.

**TABLE SM1 |** Morphological and geographic data for *Crotalus durissus* specimens included in the study, organized by voucher specimen number, sex (F = female, M = male), snout-vent length (SVL, mm), tail length (TL, mm), head length (HL, mm), head width (HW, mm), latitude (Lat), longitude (Long), and collection year for each specimen. Geographic coordinates are in decimal degrees. IBSP = Instituto Butantan; MZUESC = Museu de Zoologia da Universidade Estadual de Santa Cruz; MZUFV = Museu de Zoologia João Moojen; MNRJ = Museu Nacional do Rio de Janeiro; MZUSP = Museu de Zoologia da Universidade de São Paulo; ZUFMS = Coleção Zoológica da Universidade Federal de Mato Grosso do Sul.

| **Voucher** | **Sex** | **SVL** | **TL** | **HL** | **HW** | **Lat** | **Long** | **Data Collection** |
| --- | --- | --- | --- | --- | --- | --- | --- | --- |
| IBSP 11192 | F | 810 | 65 | 48 | 45 | -20.71 | -48.52 | 30 August 1947 |
| IBSP 11212 | F | 1121 | 78 | 60 | 50 | -23.22 | -50.82 | 02 September 1947 |
| IBSP 1170 | F | 1100 | 89 | 59 | 40 | -9.529 | -35.91 | 1916 |
| IBSP 11931 | F | 919 | 61 | 43 | 27 | -27.88 | -50.29 | 17 May 1948 |
| IBSP 12505 | M | 1030 | 115 | 55 | 45 | -27.88 | -50.29 | 25 March 1949 |
| IBSP 12680 | F | 965 | 85 | 55 | 43 | -18.63 | -48.18 | July 1949 |
| IBSP 1388 | F | 1270 | 89 | 71 | 69 | -8.35 | -36.69 | October 1917 |
| IBSP 14937 | M | 710 | 70 | 49 | 38 | -0.925 | -49.64 | November 1952 |
| IBSP 1537 | M | 970 | 103 | 56 | 46 | -5.146 | -39.18 | January 1918 |
| IBSP 15401 | F | 820 | 60 | 46 | 39 | -0.925 | -49.64 | 09 June1953 |
| IBSP 1616 | F | 1375 | 129 | 87 | 50 | -8.002 | -34.95 | July 1918 |
| IBSP 17726 | M | 960 | 117 | 59 | 43 | -0.925 | -49.64 | 05 December 1959 |
| IBSP 18331 | F | 1185 | 96 | 49 | 25 | -24.14 | -53.08 | 27 November 1958 |
| IBSP 18652 | M | 1080 | 115 | 59 | 49 | -0.925 | -49.64 | 05 December 1958 |
| IBSP 20438 | F | 975 | 81 | 57 | 41 | -15.78 | -47.87 | 23 June1961 |
| IBSP 23991 | M | 1305 | 182 | 71 | 50 | -9.368 | -36.24 | 29 May 1964 |
| IBSP 31179 | F | 915 | 75 | 55 | 42 | -9.39 | -40.5 | 03 June 1970 |
| IBSP 32166 | F | 853 | 58 | 52 | 37 | -23.07 | -55.63 | 25 February 1971 |
| IBSP 32929 | M | 784 | 82 | 46 | 40 | -23.07 | -55.63 | November 1971 |
| IBSP 33096 | M | 928 | 98 | 41 | 30 | -15.62 | -56.09 | 11 May 1972 |
| IBSP 33198 | M | 805 | 84 | 50 | 39 | -23.07 | -55.63 | 05 April 1972 |
| IBSP 33231 | M | 715 | 76 | 40 | 30 | -23.07 | -55.63 | 05 April 1972 |
| IBSP 33801 | M | 1185 | 147 | 66 | 55 | -6.974 | -37.34 | 21 Feburary 1973 |
| IBSP 38054 | F | 940 | 75 | 50 | 40 | -20.43 | -51.32 | 02 July 1973 |
| IBSP 38056 | M | 845 | 90 | 47 | 40 | -20.43 | -51.32 | 02 July 1973 |
| IBSP 40398 | M | 971 | 92 | 55 | 39 | -29.34 | -50.78 | 05 March 1974 |
| IBSP 48962 | M | 1085 | 120 | 62 | 50 | -21.18 | -50.57 | 23 January 1978 – 22 October 1985 |
| IBSP 52879 | F | 865 | 57 | 50 | 40 | -22.18 | -43.77 | 20 May 1986 |
| IBSP 54509 | M | 1110 | 110 | 60 | 45 | -18 | -46.91 | 29 July 1991 |
| IBSP 54540 | M | 835 | 100 | 50 | 40 | -5.478 | -43.7 | 08 November 1990 |
| IBSP 55452 | F | 957 | 50 | 36 | 25 | -22.78 | -51.55 | 03 June 1994 |
| IBSP 55460 | F | 968 | 53 | 40 | 25 | -22.78 | -51.55 | 03 June 1994 |
| IBSP 55531 | F | 810 | 50 | 45 | 35 | -22.18 | -43.77 | 26 July 1994 |
| IBSP 56899 | M | 1000 | 129 | 61 | 44 | -5.512 | -47.47 | 17 March 1997 |
| IBSP 56903 | F | 955 | 78 | 58 | 42 | -5.512 | -47.47 | 17 March 1997 |
| IBSP 58781 | F | 850 | 65 | 44 | 42 | -22.24 | -47.82 | 22 September 1998 |
| IBSP 59129 | M | 905 | 102 | 53 | 39 | -23.98 | -53.74 | 08 October 1998 |
| IBSP 59260 | F | 810 | 57 | 55 | 45 | -14.04 | -52.15 | 13 November 1998 |
| IBSP 61192 | M | 1028 | 104 | 41 | 21 | -22.65 | -52.85 | 18 March – 09 June 1999 |
| IBSP 61202 | F | 980 | 80 | 37 | 28 | -23.48 | -51.82 | 18 March – 09 June 1999 |
| IBSP 64041 | M | 840 | 88 | 68 | 38 | -14.22 | -42.81 | 16 June2001 |
| IBSP 64152 | M | 710 | 46 | 60 | 39 | -14.22 | -42.81 | 04 September2001 |
| IBSP 67177 | M | 795 | 97 | 50 | 38 | -22.24 | -47.82 | 07 May 2002 |
| IBSP 67984 | F | 800 | 70 | 55 | 45 | -14.04 | -52.15 | 08 November 2002 |
| IBSP 69224 | F | 935 | 65 | 41 | 22 | -13.25 | -53.08 | 06 June – 11 July 2003 |
| IBSP 70666 | M | 775 | 75 | 45 | 33 | -23.98 | -53.74 | 13 April 2004 |
| IBSP 78967 | M | 713 | 72 | 44 | 35 | -21.9 | -45.08 | 17 December 2010 – 14 January 2011 |
| IBSP 78968 | F | 870 | 61 | 50 | 40 | -21.9 | -45.08 | 17 December 2010 – 14 January 2011 |
| IBSP 78969 | M | 850 | 75 | 48 | 30 | -21.9 | -45.08 | 17 December 2010 - 14 January 2011 |
| IBSP 81632 | F | 920 | 55 | 50 | 36 | -23.3 | -47.27 | 17 January – 07 February 2012 |
| IBSP 81637 | M | 748 | 74 | 48 | 34 | -23.3 | -47.27 | 17 January – 07 February 2012 |
| IBSP 85373 | M | 950 | 104 | 57 | 43 | -22.37 | -49.06 | 12 February – 02 April 2014 |
| IBSP 85385 | F | 880 | 57 | 52 | 43 | -22.11 | -50.18 | 29 March 2014 |
| IBSP 86513 | M | 935 | 98 | 50 | 50 | -22.11 | -50.18 | 29 January 2015 |
| IBSP 87439 | F | 925 | 60 | 41 | 27 | -21.71 | -57.87 | 14 September 2015 |
| IBSP 89848 | F | 1094 | 65 | 45 | 26 | -26.54 | -52.56 | 16 February – 20 March 2018 |
| IBSP 90451 | M | 815 | 84 | 50 | 35 | -5.6 | -36.61 | 23 November 2023 |
| IBSP 90453 | M | 849 | 74 | 42 | 35 | -5.6 | -36.61 | 23 November 2023 |
| IBSP 9794 | F | 820 | 68 | 50 | 42 | -9.646 | -35.7 | 19 January 1937 |
| MNRJ 10156 | M | 801 | 83 | 45 | 40 | -22.84 | -46.94 | 28 November 2003 |
| MNRJ 14209 | F | 905 | 60 | 50 | 40 | -21.9 | -45.08 | June 2006 |
| MNRJ 15045 | F | 954 | 80 | 50 | 45 | -17.74 | -46.17 | 05 February 2007 |
| MNRJ 15335 | F | 915 | 65 | 55 | 42 | -17.74 | -46.17 | 2006 |
| MNRJ 15353 | M | 955 | 82 | 50 | 32 | -17.74 | -46.17 | December 2006 |
| MNRJ 17216 | M | 1005 | 105 | 58 | 49 | -17.74 | -46.17 | January – September 2008 |
| MNRJ 21637 | M | 779 | 86 | 45 | 32 | -21.96 | -43.31 | 06 March 2012 |
| MNRJ 24680 | F | 840 | 70 | 45 | 35 | -14.93 | -59.95 | 29 November 2013 |
| MNRJ 25971 | M | 775 | 89 | 39 | 39 | -21.77 | -43.43 | 09 March 2016 |
| MNRJ 26352 | M | 820 | 76 | 44 | 30 | -22.06 | -43.2 | 28 November 2016 |
| MNRJ 26694 | M | 791 | 80 | 41 | 35 | -22.11 | -43.17 | 29 August 2016 |
| MNRJ 2994 | F | 1090 | 69 | 51 | 32 | -13.25 | -43.42 | 23 March 1942 |
| MNRJ 7133 | M | 1105 | 95 | 51 | 36 | -17.28 | -48.28 | 1999 |
| MNRJ 8597 | M | 800 | 90 | 49 | 34 | -18.26 | -46.35 | 18 April 1990 |
| MZUESC 10043 | F | 885 | 66 | 55 | 47 | -13.44 | -41.81 | 16 November 2010 – 27 December 2011 |
| MZUESC 10044 | F | 1059 | 69 | 60 | 52 | -13.44 | -41.81 | 16 November 2010 – 27 December 2011 |
| MZUESC 10075 | M | 796 | 91 | 50 | 35 | -13.15 | -41.79 | 14 November 2010 – 29 December 2011 |
| MZUESC 10136 | M | 1015 | 113 | 61 | 48 | -14.52 | -40.37 | 06 October 2011 – 11 January 2012 |
| MZUESC 10137 | M | 941 | 115 | 54 | 32 | -14.52 | -40.37 | 06 October 2011 – 11 January 2012 |
| MZUESC 10867 | F | 1050 | 101 | 59 | 49 | -13.47 | -40.22 | 22 August 2012 |
| MZUESC 10984 | M | 792 | 95 | 56 | 39 | -13.44 | -41.81 | 28 December 2011 – 20 September 2012 |
| MZUESC 11247 | F | 871 | 76 | 50 | 35 | -11.55 | -41.15 | 06 June – 18 November 2012 |
| MZUESC 12603 | M | 751 | 83 | 50 | 30 | -12.3 | -40.49 | 19 November 2012 – 21 December 2013 |
| MZUESC 14041 | M | 1005 | 127 | 58 | 47 | -13.4 | -44.18 | 28 August 2013 – 31 August 2014 |
| MZUESC 14767 | M | 873 | 90 | 50 | 31 | -11.55 | -41.15 | 28 August 2014 – 02 October 2015 |
| MZUESC 14816 | F | 940 | 76 | 45 | 35 | -11.55 | -41.15 | 28 August 2014 – 02 October 2015 |
| MZUESC 20814 | F | 842 | 64 | 51 | 41 | -13.44 | -41.81 | 03 September 2017 – 06 April 2019 |
| MZUESC 20817 | M | 1075 | 124 | 58 | 40 | -13.44 | -41.81 | 03 September 2017 – 06 April 2019 |
| MZUESC 20857 | F | 1031 | 59 | 57 | 36 | -13.44 | -41.81 | 04 September 2017 – 04 April 2019 |
| MZUESC 20865 | M | 1005 | 121 | 55 | 45 | -13.44 | -41.81 | 04 September 2017 – 04 April 2002 |
| MZUESC 2649 | F | 825 | 67 | 54 | 40 | -13.44 | -41.81 | 22 November 2001 – 26 June 2002 |
| MZUESC 8917 | M | 1103 | 145 | 60 | 45 | -13.47 | -40.22 | 18 May 2008 – 22 September 2010 |
| MZUESC 9103 | F | 1015 | 85 | 60 | 40 | -14.52 | -40.37 | 11 June – 24 November 2010 |
| MZUESC 9139 | M | 985 | 116 | 52 | 45 | -13.44 | -41.81 | 20 December 2009 – 16 November 2010 |
| MZUESC 9209 | M | 1250 | 125 | 66 | 42 | -13.85 | -40.03 | 21 December 2010 |
| MZUESC 9656 | M | 1045 | 125 | 61 | 40 | -14.52 | -40.37 | 24 November 2010 – 20 August 2011 |
| MZUESC 9658 | M | 915 | 120 | 52 | 40 | -14.52 | -40.37 | 01 June – 20 August 2011 |
| MZUESC 9706 | M | 775 | 85 | 45 | 38 | -11.55 | -41.15 | 18 July 2010 – 15 October 2011 |
| MZUESC 9724 | M | 781 | 94 | 49 | 37 | -11.55 | -41.15 | 18 July 2010 – 15 October 2011 |
| MZUESC 9727 | M | 994 | 110 | 50 | 36 | -11.55 | -41.15 | 18 July 2010 – 15 October 2011 |
| MZUFV 1198 | F | 851 | 93 | 44 | 27 | -21.76 | -43.35 | 01 November 2004 |
| MZUFV 196 | M | 930 | 100 | 45 | 31 | -18.6 | -46.52 | 05 April1979 |
| MZUFV 197 | F | 899 | 74 | 43 | 27 | -19.89 | -44.43 | 16 June 1983 |
| MZUFV 199 | M | 860 | 121 | 45 | 27 | -19.89 | -44.43 | 25 August 1983 |
| MZUFV 2572 | M | 950 | 101 | 47 | 31 | -20.76 | -42.89 | 22 February 2019 |
| MZUFV 2758 | M | 1021 | 111 | 48 | 29 | -20.76 | -42.88 | 10 February 2020 |
| MZUFV 2834 | F | 810 | 55 | 41 | 31 | -22.05 | -42.52 | 27 March 2022 |
| MZUFV 3024 | M | 1020 | 120 | 55 | 36 | -15.91 | -46.1 | 24 March 2023 |
| MZUFV 385 | M | 801 | 91 | 43 | 28 | -20.67 | -43.09 | 27 April 1987 |
| MZUFV 452 | M | 924 | 105 | 45 | 28 | -20.65 | -42.86 | 27 March 1991 |
| MZUFV 700 | M | 779 | 90 | 41 | 21 | -21.7 | -43.39 | 12 June 1986 |
| MZUFV 704 | M | 806 | 101 | 40 | 27 | -21.65 | -43.41 | 12 June 1986 |
| MZUFV 706 | M | 825 | 91 | 43 | 25 | -21.84 | -43.79 | 12 March 1986 |
| MZUFV 853 | M | 750 | 85 | 39 | 21 | -20.26 | -42.04 | 12 June 1986 |
| MZUFV 856 | M | 975 | 100 | 46 | 28 | -20.26 | -42.03 | 12 June 1986 |
| MZUFV 891 | F | 850 | 69 | 43 | 27 | -20.27 | -42.05 | 12 June 1996 |
| MZUSP 10010 | M | 826 | 105 | 52 | 44 | 3.431 | -61.67 | 28 September 1989 |
| MZUSP 10016 | M | 1086 | 142 | 63 | 53 | 3.431 | -61.67 | October 1985 |
| MZUSP 10075 | M | 721 | 80 | 45 | 44 | -10.79 | -42.82 | 09 February 1989 |
| MZUSP 10098 | F | 1221 | 112 | 68 | 55 | 1.707 | -61.31 | 21 February 1990 |
| MZUSP 10732 | M | 854 | 94 | 52 | 45 | 2.835 | -60.68 | 10 November 1993 |
| MZUSP 10918 | M | 817 | 95 | 47 | 45 | 3.311 | -62.35 | 10 December 1994 |
| MZUSP 9190 | F | 944 | 87 | 54 | 49 | 2.835 | -60.68 | January 1986 |
| MZUSP 9193 | M | 720 | 84 | 44 | 33 | 2.835 | -60.68 | January 1986 |
| MZUSP 9307 | M | 1205 | 150 | 82 | 64 | 3.431 | -61.67 | 04 July 1986 |
| MZUSP 9958 | F | 892 | 79 | 59 | 47 | 2.835 | -60.68 | 06 August 1989 |
| ZUFMS 00264 | M | 886 | 94 | 43 | 29 | -19.01 | -57.65 | 01 November 1991 |
| ZUFMS 01883 | M | 1000 | 112 | 46 | 30 | -19.92 | -54.36 | 15 August 1986 |
| ZUFMS 01887 | M | 837 | 86 | 42 | 21 | -19.73 | -51.93 | 06 October 1986 |
| ZUFMS 01891 | M | 801 | 83 | 39 | 26 | -20.44 | -54.65 | 10 July 1987 |
| ZUFMS 02849 | M | 1061 | 105 | 48 | 26 | -21.8 | -54.55 | 02 December 1986 |
| ZUFMS 03170 | F | 1120 | 75 | 52 | 32 | -20.48 | -55.81 | 16 September 1986 |
| ZUFMS 03174 | F | 895 | 59 | 46 | 25 | -21.68 | -54.92 | 07 April 1986 |

**TABLE SM2 |** Loadings of morphological variables on the first two principal components. PC1 explains 65.9% of total morphological variation and represents a general body size axis, with strong positive loadings across all traits. SVL = snout-vent length; TL = tail length; HL = head length; HW = head width.

| **Variable** | **Dim.1** | **Dim.2** |
| --- | --- | --- |
| SVL | 0.8042 | 0.3169 |
| TL | 0.7206 | 0.5440 |
| HL | 0.9217 | -0.2305 |
| HW | 0.7886 | -0.5510 |

**TABLE SM3 |** Loadings of the 19 bioclimatic variables (WorldClim v2.1) on the first two axes of the climatic PCA. Dim.1 explains 48.1% of total climatic variation and primarily reflects a temperature-related gradient.

| **Climatic Variables** | **Dim.1** | **Dim.2** |
| --- | --- | --- |
| Bio01 – Annual Mean Temperature | 0.94617489 | -0.08810239 |
| Bio02 – Mean Diurnal Range | -0.40601359 | -0.22383348 |
| Bio03 – Isothermality | 0.79690267 | -0.12346096 |
| Bio04 – Temperature Seasonality | -0.76194885 | 0.28107645 |
| Bio05 – Max Temperature of Warmest Month | 0.77738650 | -0.06722275 |
| Bio06 – Min Temperature of Coldest Month | 0.96334526 | -0.02167286 |
| Bio07 – Temperature Annual Range | -0.67416436 | -0.02551616 |
| Bio08 – Mean Temperature of Wettest Quarter | 0.76313127 | -0.17187206 |
| Bio09 – Mean Temperature of Driest Quarter | 0.97110714 | -0.02896022 |
| Bio10 – Mean Temperature of Warmest Quarter | 0.84167942 | 0.02332185 |
| Bio11 – Mean Temperature of Coldest Quarter | 0.97946384 | -0.12891628 |
| Bio12 – Annual Precipitation | 0.41277257 | 0.73866243 |
| Bio13 – Precipitation of Wettest Month | 0.56408299 | 0.34118527 |
| Bio14 – Precipitation of Driest Month | -0.08640988 | 0.91893347 |
| Bio15 – Precipitation Seasonality | 0.36423054 | -0.71551507 |
| Bio16 – Precipitation of Wettest Quarter | 0.55968907 | 0.36981940 |
| Bio17 – Precipitation of Driest Quarter | -0.09485982 | 0.91750504 |
| Bio18 – Precipitation of Warmest Quarter | -0.52691796 | 0.18734860 |
| Bio19 – Precipitation of Coldest Quarter | 0.67499700 | 0.66418078 |

**TABLE SM4** | Description of the 19 bioclimatic variables extracted from WorldClim database version 2.1.

| **Variable Code** | **Description** | **Unit** |
| --- | --- | --- |
| BIO01 | Annual Mean Temperature | °C |
| BIO02 | Mean Diurnal Range (Mean of monthly (max temp - min temp)) | °C |
| BIO03 | Isothermality (BIO2/BIO7) (×100) | Dimensionless |
| BIO04 | Temperature Seasonality (standard deviation ×100) | Dimensionless |
| BIO05 | Max Temperature of Warmest Month | °C |
| BIO06 | Min Temperature of Coldest Month | °C |
| BIO07 | Temperature Annual Range (BIO5-BIO6) | °C |
| BIO08 | Mean Temperature of Wettest Quarter | °C |
| BIO09 | Mean Temperature of Driest Quarter | °C |
| BIO10 | Mean Temperature of Warmest Quarter | °C |
| BIO11 | Mean Temperature of Coldest Quarter | °C |
| BIO12 | Annual Precipitation | mm |
| BIO13 | Precipitation of Wettest Month | mm |
| BIO14 | Precipitation of Driest Month | mm |
| BIO15 | Precipitation Seasonality (Coefficient of Variation) | Dimensionless |
| BIO16 | Precipitation of Wettest Quarter | mm |
| BIO17 | Precipitation of Driest Quarter | mm |
| BIO18 | Precipitation of Warmest Quarter | mm |
| BIO19 | Precipitation of Coldest Quarter | mm |

**TABLE SM5** | Multivariate (MANOVA) and univariate (ANOVA) tests of sexual dimorphism in *Crotalus durissus*. The MANOVA indicates a significant overall effect of sex on multivariate morphology (Wilks’ *λ* = 0.319, *F*₄,₁₂₇ = 67.91, *p* < 0.001). Univariate ANOVAs show that tail length (TL) differs significantly between sexes, whereas snout–vent length (SVL), head length (HL), and head width (HW) do not show significant differences.

| **MANOVA** | | | | | |
| --- | --- | --- | --- | --- | --- |
| **Test** | **Wilks’ *λ*** | ***F*** | ***df1*** | ***df2*** | ***P* value** |
| Sex effect | 0.31858 | 67.91 | 4 | 127 | <0.001 |
| **ANOVA** | | | | | |
| **Trait** | ***F*** | ***df1*** | ***df2*** | ***P* value** | |
| SVL | 3.1549 | 1 | 130 | 0.07804 | |
| TL | 68.312 | 1 | 130 | <0.001 | |
| HL | 0.1108 | 1 | 130 | 0.7398 | |
| HW | 0.5677 | 1 | 130 | 0.4525 | |
